# Electroencephalography Patterns of Subacute Sclerosing Panencephalitis

**DOI:** 10.7759/cureus.15728

**Published:** 2021-06-17

**Authors:** Saad Ali, Harwindar Kumar, Shakir Ullah, Mian Ayaz U Haq, Nusrat G Gul, Jasvindar Kumar

**Affiliations:** 1 Neurology, Lady Reading Hospital Medical Teaching Institute Peshawar, Peshawar, PAK; 2 Pharmacology, Khyber Medical University, Peshawar, PAK; 3 Neurophysiology, Lady Reading Hospital Medical Teaching Institute Peshawar, Peshawar, PAK; 4 Internal Medicine, Khyber Teaching Hospital, Peshawar, PAK

**Keywords:** electroencephalography, subacute sclerosing panencephalitis, dyken’s criteria, complication of measles, neurodegenerative disorder

## Abstract

Introduction and background: This article explores the electroencephalography (EEG) pattern in patients of suspected subacute sclerosing panencephalitis (SSPE) visiting Lady Reading Hospital, Peshawar. Pakistan has a huge deficit of research culture, and limited studies are done on this topic. This study concluded that a typical pattern is the most common EEG pattern, although atypical and normal EEG patterns were also observed. It is worth inclusion into the existing literature and may be used for future literature review of similar studies done elsewhere in Pakistan to give a wider perspective comprised of a larger sample size - integrating* *all studies.

Objective: To determine the frequency of different patterns of EEG (typical, atypical, and normal) in SSPE patients.

Methodology: Seventy-seven patients of both male and female genders between ages one to 20 years, who were diagnosed with SSPE, were included in the study. Dyken’s criteria were used to diagnose the patients. A prior history of previous measles infection with signs and symptoms suggestive of SSPE and positive anti-measles IgG antibodies in the cerebrospinal fluid (CSF) was found in all the patients included in the study. Besides this, typical EEG patterns and raised CSF globulin levels were also used for confirmation of the diagnosis. All the patients fulfilling the above criteria and presenting to the neurology department of Lady Reading Hospital, Peshawar, from February 1, 2019, to November 30, 2019, were included in the study. All the patients underwent the EEG monitoring in the same EEG laboratory and were reported by the same consultant with careful exclusion of any artifacts during the study.

Result: There were 59 (76.62%) males and 18 (23.37%) females. The mean age was 15 ± 8.6 years, and the mean duration of symptoms was 4.79 ± 1.68 months. EEG was normal in 14 (18.18%) patients, while 63 (81.81%) patients had an abnormal EEG pattern, with a majority of 53 (84.12%) patients showing periodic delta wave complexes. Only 10 (15.87%) patients showed atypical patterns.

Conclusion: Almost all the patients of SSPE showed periodic high-amplitude delta waves complexes, which usually occurs in patients with a disease duration of more than four months. However, further studies with a large sample size are needed for the confirmation of this observation.

## Introduction

Subacute sclerosing panencephalitis (SSPE) is a chronic neurodegenerative disease that is a post-infection sequela of measles [1]. The estimated frequency of SSPE post measles in the United States is 8.5 per million cases of measles [2]. In the developed world, measles and its complications are rare, but recent evidence suggests that it is more common than previously thought. The incidence of measles was noted to be one in 1367 and one in 609 in children aged <five years and <12 months, respectively. Different studies from different parts of the world suggested the incidence varies from one in 700 to one in 25,000 in the patients of measles [3-6].

SSPE usually presents with the symptoms of motor involvement such as jerking movements, muscle spasms, ataxia, tremor, seizure, etc. [7]. However, several atypical presentations are also noted [8-11]. Post-measles neurological complications include acute encephalitis, measles inclusion body encephalitis, acute disseminated encephalomyelitis (ADEM), and SSPE. SSPE has a different time and course than ADEM [12]. SSPE is quite a fatal disease in developing countries mainly due to lack of early diagnosis and no proper treatment. Some type of medical treatment in the form of isoprinosine, ribavirin, and interferon-alpha can be offered to the patients who are diagnosed at an early stage of the disease. Various diagnostic tools such as measles antibody detection in cerebrospinal fluid (CSF) and serum, neuroimaging, and EEG are routinely used for the investigation of SSPE. Among these, EEG is the simplest, non-invasive, and cheapest investigation with easy availability.

The EEG findings of SSPE are usually shown to be the periodic complexes that are high-amplitude delta waves and are repeated every four to seven seconds [12]. This EEG pattern occurs in almost all the patients at some stage of the disease [12]. Some atypical EEG patterns, which includes asymmetric periodic complexes, spike and slow waves pattern, and paroxysmal rhythmic delta activity between periodic complexes, are also seen in this disease. The long-term video-split EEG has improved the early diagnosis of this disease.

This study is designed to evaluate the cases of SSPE for the different patterns of EEG. In developing countries like Pakistan where measles and its consequences are still not only prevalent but also quite fatal, the knowledge of different EEG patterns will help in the early diagnosis of the patients.

## Materials and methods

Patients from both genders aged between one and 20 years who were diagnosed with SSPE were enrolled in the study. These patients presented between February 1, 2019, to November 30, 2019, to the neurology department of Lady Reading Hospital, Peshawar. All the patients were diagnosed with SSPE using Dyken’s criteria.

Patients with a family history of seizure, neurological problems, and meningeal signs were excluded from the study. Duration of symptoms was noted since the motor involvement. The diagnosis of SSPE was based on the history of prior measles infection, clinical features, serum and CSF measles antibody levels, CSF immunoglobulin levels, and neuroimaging. Most of the patients were having mood swings and personality changes followed by focal neurological features such as jerking movement, muscle spasms, seizure, loss of vision, etc. Neuroimaging was normal in some of the patients, but the majority had occipital white matter changes. Serum and CSF anti-measles antibody were sent to the lab, and those with anti-measles IgG antibodies in cerebrospinal fluid were taken as positive. All the patients underwent the EEG monitoring in the same EEG lab and were reported by the same consultant with careful exclusion of any artifacts during the study.

Periodic delta wave complexes were composed of slow waves in the delta range with a high amplitude (200-500 mV), symmetrical and bisynchronous. These waves were repeatedly seen every 4-10 seconds. The other atypical patterns observed were asymmetric periodic complexes, spikes, and spike-slow wave complexes.

Data were analyzed through Statistical Package for the Social Sciences (SPSS) version 20 (IBM Corp., Armonk, NY, USA). The percentages, frequency, mean, standard deviation (SD), and p values were calculated and presented in tables in this article.

## Results

Out of 77 patients, 59 (76.62%) were males and 18 (23.37%) were females. The mean age was 15 ± 8.6 years, and the mean duration of symptoms was 4.79 ± 1.68 months (Table 1).

**Table 1:** Baseline characteristics (n = 77)

		Values
Gender	Male	59 (76.62%)
	Female	18 (23.37%)
Age (years)		15 ± 8.6
Duration of symptoms (months)		4.79 ± 1.68

**Figure 1:**
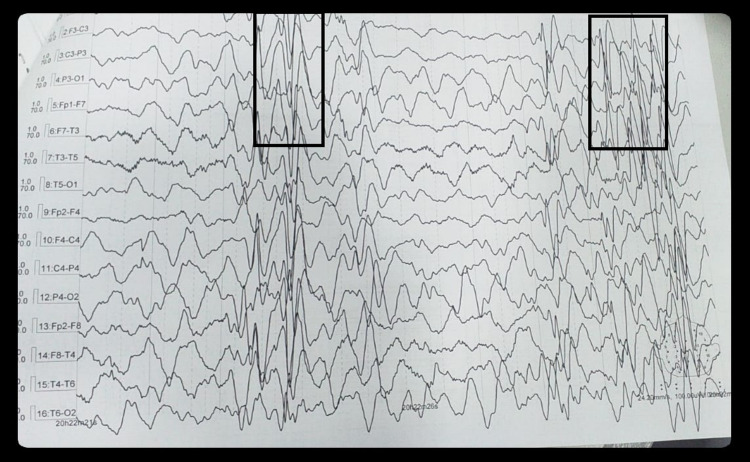
Symmetrical periodic slow wave complexes (typical pattern)

**Figure 2:**
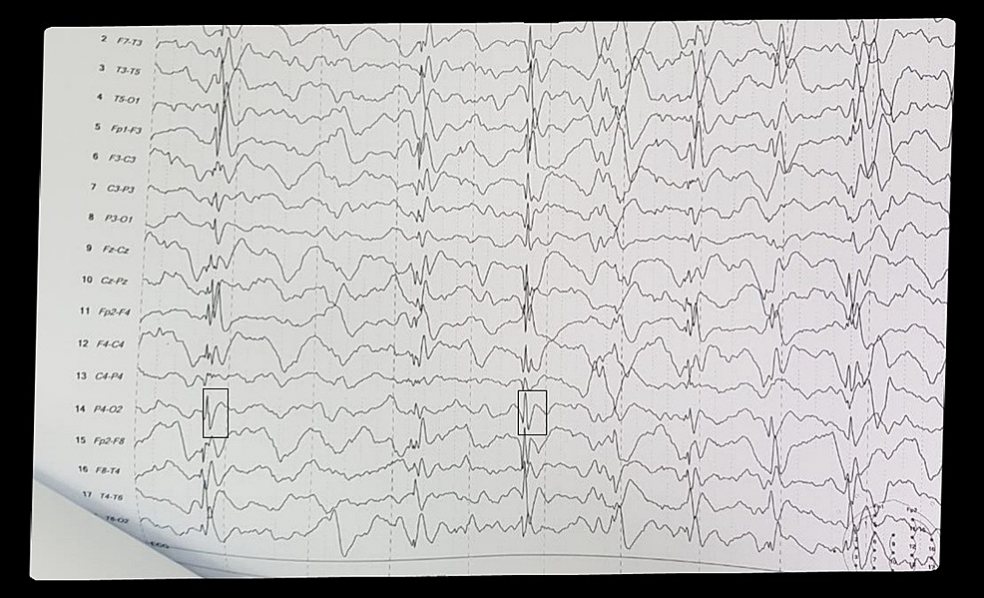
Spike and slow waves (atypical pattern)

EEG was normal in 14 (18.18%) patients, while 63 (81.81%) patients had an abnormal EEG finding (Table 2). Among these, 53 (84.12%) patients showed typical periodic slow wave complexes (Figure 1), while 10 patients (15.18%) had atypical patterns (Table 3). This included asymmetric periodic complexes, spike, and slow waves pattern (Figure 2) as well as paroxysmal rhythmic delta activity between periodic complexes as 60%, 30%, and 10%, respectively (Table 4).

**Table 2:** EEG findings (n = 77) EEG, Electroencephalography.

EEG findings	Number of patients
Abnormal EEG	63 (81.81%)
Normal EEG	14 (18.18%)

**Table 3:** Abnormal EEG findings (n = 63) EEG, Electroencephalography.

EEG findings	Number of patients
Typical symmetrical periodic slow wave complexes	53 (84.12%)
Atypical patterns	10 (15.87%)

**Table 4:** Types of atypical patterns included (n = 10)

Types of atypical patterns	Number of patients
Asymmetric periodic complexes	6 (60%)
Spike and slow waves pattern	3 (30%)
Paroxysmal rhythmic delta activity between periodic complexes	1 (10%)

The duration of symptoms affected the patterns of EEG. There was a positive correlation between the duration of symptoms and the occurrence of periodic delta waves (typical pattern) (p-value = 0.125) (Table 5).

**Table 5:** Association of demographic features and duration of symptoms against EEG pattern (n = 77) EEG, Electroencephalography.

		EEG			
		Normal EEG	Periodic delta wave complexes	Atypical pattern	P values
Gender	Male	11 (18.64%)	32 (54.23%)	16 (27.11%)	0.641
	Female	3 (16.66%)	7 (38.88%)	8 (44.44%)	
Age groups	<12-years old	11 (21.56%)	28 (45.09%)	17 (33.33%)	0.154
	>12-years old	3 (14.28%)	10 (47.61%)	8 (38.09%)	
Duration groups	<4 months of symptoms	4 (5.19%)	13 (16.88%)	4 (5.19%)	0.125
	>4 months of symptoms	10 (12.98%)	40 (51.94%)	6 (7.79%)	

In our results, SSPE was found more in males (76.62%) as compared to females (23.37%) with a ratio of 3.27 to 1.

## Discussion

Measles is the most contagious virus yet known, and it is still an important cause of childhood mortality and blindness in developing countries as well as sporadic outbreaks in the industrialized nation [13]. Vaccination against measles is effective, cost-effective, and safe [14]. It is almost six decades since vaccination for the prevention of measles has been licensed [15]. However, most of the population in developing and under-developed countries remained unvaccinated because of which measles and its complications are still seen in clinical practice. It requires a high level of population immunity to interrupt the transmission of measles; therefore, it is difficult to eliminate in the high-population density areas [16].

Dyken's criteria are often used as the diagnostic tool for SSPE; this includes clinical history, elevated CSF measles antibody titers, typical EEG pattern, increased CSF immunoglobulin G (IgG), and brain biopsy.

Measles causes four major central nervous system (CNS) syndromes: acute encephalitis, post-viral encephalomyelitis, measles inclusion body encephalitis, and subacute sclerosing panencephalitis (SSPE) [17]. Symptoms of SSPE typically present about eight to 11 years of post-measles infection [18]. The chances of SSPE are higher if measles is acquired earlier in life [2,6]. Individuals infected with the human immunodeficiency virus (HIV) or those who are born to mothers with HIV infection might be at higher risk of developing SSPE after measles infection [19,20].

Currently, there is no treatment for SSPE, and eradication by an effective vaccination program is considered to be most beneficial and cost-effective than the medications considered for the management of SSPE [21].

SSPE is usually a life‐threatening disease with no cure, despite the development of antiviral and immunomodulatory drugs. A multicentered non‐randomized study of 98 patients with SSPE treated with isoprinosine reported increased survival for over two years in the intervention group [22]. SSPE is caused by particular mutants of the measles virus, which are often referred to as the SSPE virus [23].

In our study, we found that most patients (63 out of 77) with SSPE had an abnormal EEG, and the most common abnormality was periodic high-amplitude delta waves complexes that occurred in 53 patients (79.4%) and the atypical patterns that occurred in 10 patients (15.87%). The study done by Praveen-kumar et al. showed a typical pattern in 37 patients and atypical patterns in 21 patients [24]. Another study by Wulff et al. found periodic complexes in all patients of SSPE [25]. Our results correlated with all these studies.

There was no association between age and pattern of EEG in our study. The mean age in our study was 15 ± 8.6 years. However, maturation to posterior alpha rhythm occurs around 12 years of age; therefore, there may be some bias over here.

The duration of the disease showed a significant relation to periodic delta wave complexes as with the onset of motor symptoms. The majority (51.94%) of patients with motor symptoms after more than four months of disease onset had positive EEG for periodic high-amplitude delta waves. As the disease is quite fatal in developing countries, the cases with a longer duration from the time of onset were not seen. Other studies in which long-term duration was noted showed that after some years in disease progression the cortical damage occurs, and the EEG changes, then almost disappear [25].

The shorter duration of disease in our study was an important limitation that limited us from showing the EEG pattern in advanced disease. Another limitation was evaluation of only the type I periodic high-amplitude complexes; the other type II and type III complexes were not evaluated, and it might have merged in the atypical pattern. Furthermore, due to one institution focused, shorter duration of symptoms, and small sample size, the result cannot be generalized to all SSPE patients. For these reasons, another study with a large number of patients is needed.

## Conclusions

Almost all the patients of SSPE showed periodic high-amplitude delta waves that usually occurred in patients with a disease duration of more than four months. This study also confirms the SSPE ratio of male to female (3:1), which has been shown by other studies. In addition, the typical pattern of EEG is more common in SSPE; however, we should also keep in mind that atypical and normal patterns of EEG can also occur in SSPE. In the developing world with limited resources, EEG can be used as a good diagnostic tool for the suspected cases of SSPE.
